# Individualized luteal phase support based on serum progesterone levels in frozen-thawed embryo transfer cycles maximizes reproductive outcomes in a cohort undergoing preimplantation genetic testing

**DOI:** 10.3389/fendo.2022.1051857

**Published:** 2022-12-02

**Authors:** Bertille du Boulet, Noemie Ranisavljevic, Caroline Mollevi, Sophie Bringer-Deutsch, Sophie Brouillet, Tal Anahory

**Affiliations:** ^1^ Department of Reproductive Medicine, Montpellier University Hospital, University of Montpellier, Montpellier, France; ^2^ Institute Desbrest of Epidemiology and Public Health, Montpellier University Hospital, University of Montpellier, INSERM, Montpellier, France; ^3^ Department of Reproductive Biology-CECOS, Montpellier University Hospital, University of Montpellier, Montpellier, France; ^4^ Embryo Development Fertility Environment, University of Montpellier, INSERM 1203, Montpellier, France

**Keywords:** frozen-thawed embryo transfer (FET), preimplantation genetic testing (PGT), serum progesterone, ongoing pregnancy, hormone replacement therapy-luteal phase support

## Abstract

**Introduction:**

Low serum progesterone concentration on frozen embryo transfer (FET) day in hormone replacement therapy (HRT) cycles results in lower reproductive outcomes. Recent studies showed the efficiency of a “rescue protocol’’ to restore reproductive outcomes in these patients. Here, we compared reproductive outcomes in HRT FET cycles in women with low serum progesterone levels who received individualized luteal phase support (iLPS) and in women with adequate serum progesterone levels who underwent *in vitro* fertilization for pre-implantation genetic testing for structural rearrangements or monogenic disorders.

**Design:**

This retrospective cohort study included women (18-43 years of age) undergoing HRT FET cycles with pre-implantation genetic testing at Montpellier University Hospital between June 2020 and May 2022. A standard HRT was used: vaginal micronized estradiol (6mg/day) followed by vaginal micronized progesterone (VMP; 800 mg/day). Serum progesterone was measured after four doses of VMP: if <11ng/ml, 25mg/day subcutaneous progesterone or 30mg/day oral dydrogesterone was introduced.

**Results:**

125 HRT FET cycles were performed in 111 patients. Oral/subcutaneous progesterone supplementation concerned 39 cycles (n=20 with subcutaneous progesterone and n=19 with oral dydrogesterone). Clinical and laboratory parameters of the cycles were comparable between groups. The ongoing pregnancy rate (OPR) was 41.03% in the supplemented group and 18.60% in the non-supplemented group (p= 0.008). The biochemical pregnancy rate and miscarriages rate tended to be higher in the non-supplemented group versus the supplemented group: 13.95% versus 5.13% and 38.46% versus 15.79% (p=0.147 and 0.182 respectively). Multivariate logistic regression analysis found that progesterone supplementation was significantly associated with higher OPR ​​ (adjusted OR = 3.25, 95% CI [1.38 – 7.68], p=0.007).

**Conclusion:**

In HRT FET cycles, progesterone supplementation in patients with serum progesterone concentration <11 ng/mL after four doses of VMP significantly increases the OPR.

## Introduction

In the last decades, the number of frozen embryo transfers (FET) has increased for several reasons, such as the improvement of embryo culture conditions, the development of vitrification techniques, and the increasing trend in performing single embryo transfer. However, there is still no consensus on the most effective protocol for endometrium preparation before FET (1, 2). For reasons of programming flexibility, hormone replacement therapy (HRT) is the most commonly used protocol in FET cycles. For instance, Vinsonneau et al. showed that in nine reproductive health units in France, HRT was used in 56.5% of all FET cycles (2).

In HRT cycles, there is no corpus luteum and a sufficient exogenous progesterone supplementation is required to ensure the endometrium secretory transformation for embryo implantation and pregnancy support. Vaginal micronized progesterone (VMP) is commonly used because of its convenience and uterine first-pass effect that results in higher progesterone uterine concentrations (3). However, some studies showed that there is a large inter-individual variation in serum progesterone concentration in women receiving VMP (4) and that serum progesterone concentration is insufficient in 30% to 50% of them (5–7). Several factors might explain these variations in serum progesterone after VMP treatment. Some are associated with pharmacokinetic variations due to age, body mass index (BMI), history of low progesterone concentration in a previous FET cycle, and saturation of vaginal progesterone receptors (4, 8–10). Other implicated factors are the interval between blood sampling and last VMP intake, or sexual intercourse (4, 11). Recent studies reported a negative impact of low serum progesterone concentration in the luteal phase on reproductive outcomes in HRT cycles (6, 7, 9, 12–17). Progesterone concentration was assessed at different points of the HRT cycle (from the day before embryo transfer to the day of pregnancy test or later), but pharmacokinetic studies showed that progesterone concentration reaches a plateau within 24-36h after VMP administration (18). Therefore, sampling time should not influence the results (18). Although there is no consensus about the optimal progesterone cut-off during the luteal phase in HRT cycles, the most accepted value is ~11 ng/ml around embryo transfer time, which is used as a criterion of potential fertility in natural cycles (9, 13, 16, 19).

Few studies evaluated the effectiveness of a “rescue protocol” (i.e. supplementation, mainly 25 mg subcutaneous progesterone, from the day before or the day of embryo transfer) in case of low serum progesterone (5, 9, 20–22). Most of them reported similar reproductive outcomes in women with adequate progesterone levels and in women with supplementation and concluded that the rescue protocol restores reproductive outcomes (5, 20–22). Different forms of progesterone are available, but there is no consensus on what is the best for luteal phase support (LPS) (23–25). Due to its high bioavailability and easy use, oral dydrogesterone was proposed as an alternative to VMP and was found to be effective (reproductive outcomes) in both fresh and frozen embryo transfer cycles (26–29). However, no study assessed dydrogesterone in rescue protocols. This could be related to the fact that unlike vaginal and injectable progesterone forms, plasma dydrogesterone can only be measured with specific, not routinely used laboratory techniques (30).

The aim of our study was to assess whether a rescue protocol with 25 mg subcutaneous progesterone or 30 mg oral dydrogesterone per day can optimize the success rate of HRT FET cycles in women with serum progesterone <11ng/ml compared with women with adequate serum progesterone who underwent *in vitro* fertilization (IVF) for pre-implantation genetic testing for structural rearrangements (PGT-SR) or monogenic disorders (PGT-M).

## Material and methods

### Study design and eligibility criteria

This retrospective cohort study was performed at the fertility unit of Montpellier University Hospital from June 2020 to May 2022. The rescue protocol was introduced in June 2020 and consists in serum progesterone measurement after four doses of VMP followed by additional progesterone supplementation if the serum concentration is <11ng/ml.

Inclusion criteria were: HRT FET cycles in 18 to 43-year-old women undergoing IVF for preimplantation genetic testing in whom progesterone concentration was monitored after VMP introduction. Exclusion criteria were: cycles in patients with history of recurrent miscarriage (≥3), uterine malformation, hydrosalpinx, or cycles in which the standard luteal phase support (LPS) protocol was not strictly followed.

### HRT FET cycles and rescue protocol

HRT consisted in vaginal administration of micronized estradiol (3 mg twice/day) (Provames^®^; Merus Labs Luxco, Luxembourg) from the second day of the menstrual cycle for 14 days. Then, a vaginal ultrasound was performed to assess the endometrium thickness and appearance. If thickness was ≥7 mm and a triple-line aspect was observed, VMP (400 mg) (Progestan^®^; Besins international, Montrouge, France) was introduced in the evening and then every 12 hours (8 am and 8 pm). In some patients, a gonadotropin-releasing hormone (GnRH) agonist (Decapeptyl^®^; IPSEN Pharma, Boulogne-Billancourt, France) was administered for pituitary suppression for 1-3 months before HRT initiation.

After four doses of VMP, in the morning of the third day of progesterone intake, serum progesterone concentration was measured.

Patients with progesterone concentration <11ng/ml received supplemental progesterone introduced the same evening: either orally with dydrogesterone at a dose of 10 mg three times per day (Duphaston^®^; Mylan Medical SAS, Paris, France) or by injection with subcutaneous progesterone at a dose of 25 mg each evening (Progiron^®^; IBSA Pharma SAS, Antibes, France). In case of progesterone concentration ≥11ng/ml, no treatment adjustment was made.

The progesterone choice was left to the patients after information on the posology, method of administration, and cost (dydrogesterone is fully reimbursed in France, whereas subcutaneous progesterone costs ~60 euros per week). Patients were also informed that subcutaneous progesterone, but not oral dydrogesterone, was already used as rescue protocol in other centers with good efficacy (5, 20–22). There was no progesterone concentration cut-off leading to cycle cancellation.

**Figure 1:**
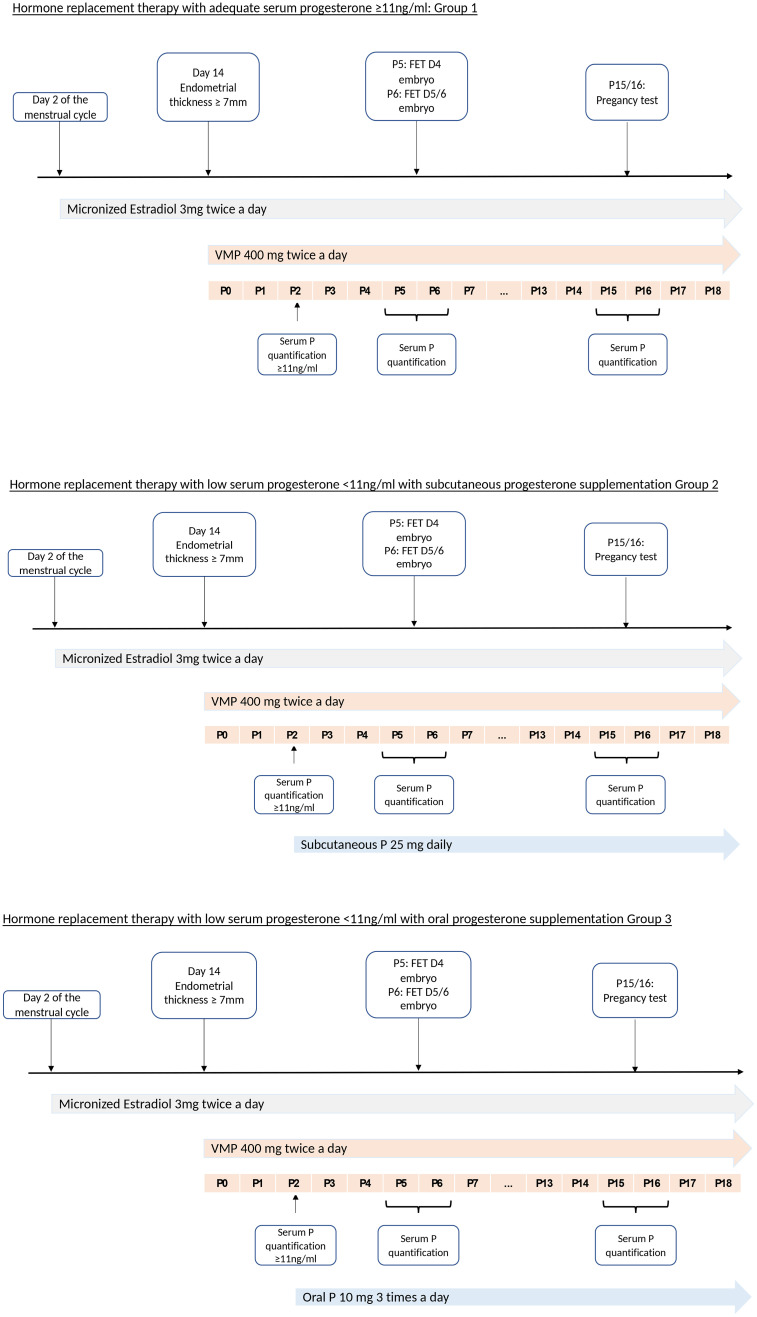
Hormone replacement therapy in each group according to the serum progesterone concentration P, progesterone; MVP, Micronized Vaginal Progesterone; P0, 1^st^ day of progesterone supplementation starting on the evening FET, frozen embryo transfer.

FET was performed under ultrasound guidance at day 5 (with day 4 embryos) or day 6 (with blastocysts) after progesterone administration. The pregnancy test was performed at day 10 or 11 after ET. Serum progesterone was quantified again on FET day and on pregnancy test day. In case of pregnancy, patients continued all treatments until week 12 of amenorrhea (**Figure 1**).

### Pre-implantation genetic testing

Embryos were issued from intracytoplasmic sperm injection cycles. In case of fresh embryo transfer, biopsy for genetic testing is performed at day 3 and embryo transfer at day 4. Remaining disease-free embryos are frozen at day 4 or at the blastocyst stage. In the case of “freeze all” strategy, two options are available: i) all embryos are frozen at day 3, and embryo biopsy and genetic testing are done after thawing, followed by FET at day 4 or at the blastocyst stage. Then, the remaining disease-free embryos are frozen again; or ii) embryo biopsy and genetic testing are done at day 3 and all disease-free embryos are frozen at day 4 or at the blastocyst stage.

### Main outcomes

The primary endpoint was to compare the ongoing pregnancy rate (OPR) between patients with serum progesterone concentration <11ng/ml after four doses of vaginal progesterone, who required and received supplementation, and patients with serum progesterone ≥11ng/ml (who, therefore, did not receive supplementation). OPR was defined as the number of viable intrauterine pregnancies detected by ultrasonography after 12 weeks of amenorrhea divided by the number of transfers.

The secondary endpoints were: i) serum progesterone concentration on embryo transfer day and pregnancy test day, ii) positive pregnancy test rate (human chorionic gonadotropin, beta subunit (beta-hCG) concentration ≥10 UI/L divided by the number of transfers), iii) implantation rate (number of intrauterine gestational sacs observed by vaginal ultrasound divided by the number of transferred embryos), iv) biochemical pregnancy rate (beta-hCG concentration ≥10 UI/L and <100UI/L divided by the number of transfers); v) clinical pregnancy rate (presence of at least one intrauterine gestational sac with fetal heartbeat divided by the number of transfers), and vi) miscarriage rate (number of spontaneous pregnancies lost before 12 weeks of amenorrhea divided by the number of intrauterine pregnancies).

### Statistical analysis

Categorical variables were reported as number of observations and frequencies and compared with the Chi-square or Fisher’s exact test. Quantitative variables were reported as mean, standard deviation, median and range and compared with the Kruskal-Wallis or Wilcoxon test.

A logistic regression model was used to identify associations between OPR and the variables of interest. For categorical variables, a reference category was chosen. For continuous variables, the odds ratio (OR) was associated with each one-unit increase. OR were reported with 95% confidence intervals (CI). Here, OR >1 means that the covariate was “favorable” and OR <1 that the covariate was “deleterious” to pregnancy.

Variables with p-values <0.10 (univariate analysis) were selected for multivariate analysis and a backward covariate selection was performed.

All tests were two-sided and p-values <0.05 were considered significant. Statistical analyses were performed with STATA 15.0 (StatCorp, College Station, TX).

## Results

### Description of the population

**Figure 2:**
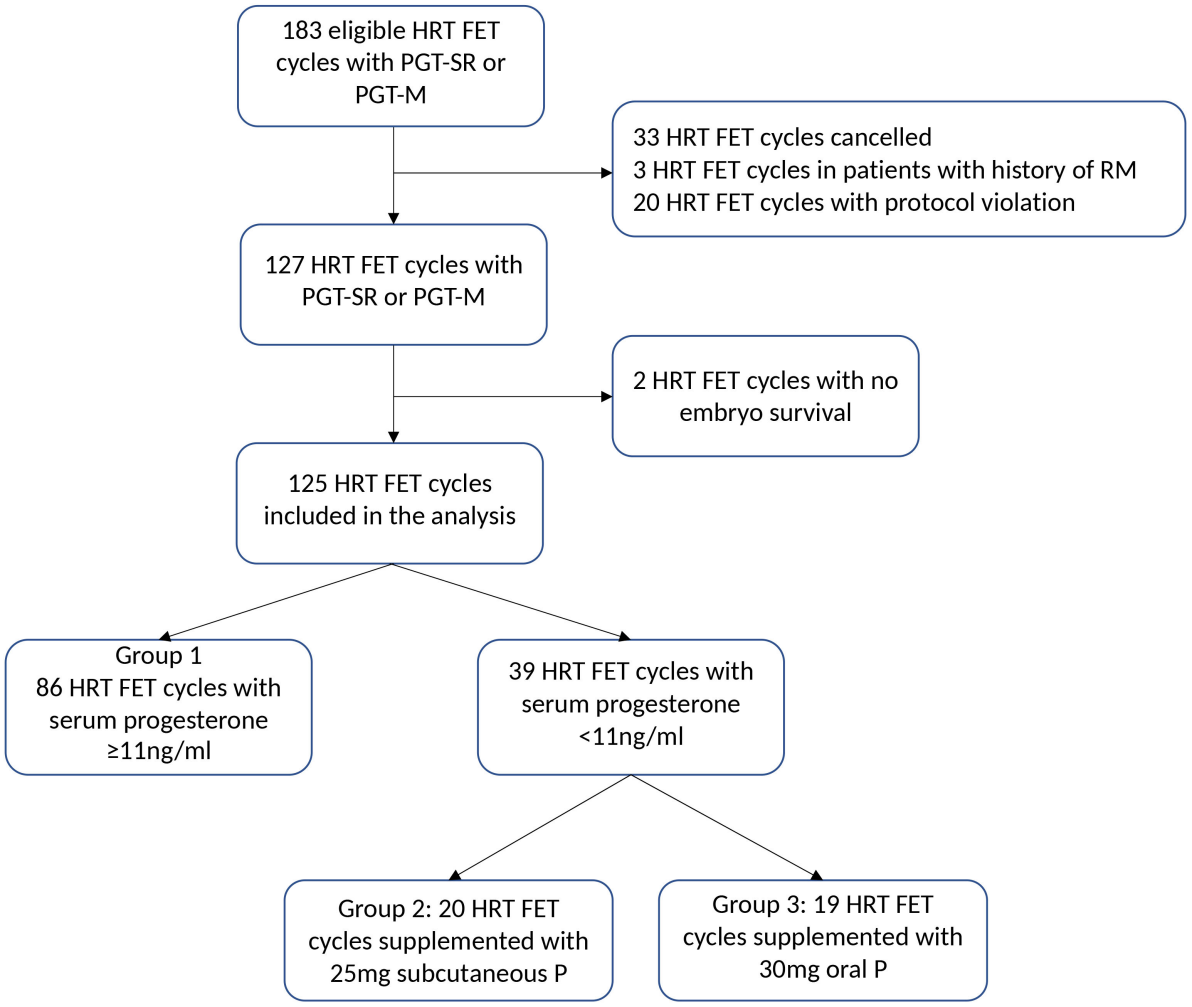
Study flowchart. HRT, hormone replacement therapy; FET, frozen embryo transfer; PGT-SR or PGCT-M, preimplantation genetic testing for structural rearrangement or monogenic disorder; RM, recurrent miscarriage; P, progesterone.

In total, 125 FET cycles with HRT performed in 111 women undergoing preimplantation genetic testing were included in the study (**Figure 2**). In 86 cycles, serum progesterone concentration after four doses of VMP was adequate (≥11ng/ml; group 1), and in 39 cycles (30.9%) was <11ng/ml, thus requiring additional progesterone (n=20 cycles with subcutaneous progesterone, group 2; and n=19 cycles with oral dydrogesterone, group 3) (**Figures 1**, **2**). Groups 2 and 3 represented the supplemented group.

The cycle and patients’ characteristics in each group (group 1 versus group 2 + 3) were comparable (**Table 1**).

**Table 1:** Demographic and HRT FET cycle features in women with and without additional progesterone supplementation.

	Group 1 (n=86)	Group 2+3 (n=39)	P-value	Group 2 (n=20)	Group 3 (n=19)
Women's age	33.21 ± 4.44	33.43 ± 4.39	0.800°	32.85 ± 4.51	34.05 ±4.29
BMI (kg/m^2^)	22.34 ± 3.08	23.33 ± 3.53	0.168°	23.55 ± 4.20	23.09 ± 2.76
Smoking					
No	69 (80.23%)	36 (92.31%)	0.116	20 (100%)	16 (84.21%)
Yes	17 (19.77%)	3 (7.69%)		0 (0%)	3 (15.79%)
Previous deliveries					
No	45 (52.33%)	16 (41.03%)	0.242*	7 (35%)	9 (47.37%)
Yes	41 (47.67%)	23 (58.97%)		13 (65%)	10 (62.63%)
Time since first attempt (months)	16.04 ± 14.65	11.08 ± 8.80	0.133°	10.85 ± 8.64	11.32 ± 9.21
AMH (ng/ml)	4.02 + 3.33	3.45 + 2.06	0.497°	4.06 ± 2.34	2.81 ± 1.52
Number of previous ET	1.16 ± 1.16	1.05 ± 1.30	0.443°	1.05 ± 1.15	1.05 ± 1.47
Number of embryos transferred in previous ET	1.53 + 1.62	1.36 + 1.83	0.370°	1.35 **+** 1.63	1.37 ± 2.06
First FET cycle					
No	40 (46.51%)	16 (41.03%)	0.568*	8 (40%)	8 (42.11%)
Yes	46 (53.49%)	23 (58.97%)		12 (60%)	11 (57.89%)
Number of aGnRH injections			0.102		
0	78 (90.7%)	35 (89.74%)		18 (90%)	17 (89.47%)
1	1 (1.16%)	3 (7.69%)		2 (10%)	1 (5.26%)
3	7 (8.14%	1 (2.56%)		0 (0%)	1 (5.26%)
Duration of estradiol before vaginal progesterone (days)	18.00 ± 3.96	18.08 ± 3.37	0.785°	18.50 ± 3.35	17.63 ± 3.43
Endometrial thickness before progesterone start (mm)	9.25 ± 1.86	9.26 + 1.86	0.996°	9.10 ± 1.96	9.43 + 1.79
Serum progesterone concentration after 4 doses (ng/ml)	16.27 ± 3.58	8.81 ± 1.35	<0.001°	9.20 ± 1.05	8.41 ± 1.53
	[11.0 ; 29.7]	[5.6; 10.9]		[7.0; 10.9]	[5.6; 10.8]
Day of embryo transfer					
Day 4	64 (74.42%)	27 (69.23%)	0,546*	15 (75%)	12 (63.16%)
Blastocyst	22 (25.58%)	12 (30.77%)		5 (25%)	7 (36.84%)
Number of embryos transferred	1.33 ± 0.47	1.38 ± 0.49	0.521°	1.55 ± 0.51	1.21 ± 0.42
Serum progesterone concentration on FET day (ng/ml)	15.46 ± 3.55	**-**	0.070	16.83 + 2.95	**-**
	[8.64 ; 25]			[12.5 ; 20.9]	
Serum progesterone concentration on pregnancy test day (ng/ml)	14.90 ± 5.28	**-**	0.457	15.03 + 3.03	**-**
	[6.2; 39.6]			[9.7; 20.4]	

Group 1: non-supplemented, Group 2: subcutaneous progesterone supplementation, Group 3: oral dydrogesterone supplementation.

P, progesterone; BMI, Body Mass Index; AMH, anti-MQIIerian hormone; ET, embryo transfer; aGnRH, gonadotropin-releasing hormone agonist; **FET,** frozen embryo transfer.

Data are presented as mean ±SD or n (%).

°Wilcoxon's test: non-supplemented group (N=86) vs supplemented groups together (2+3) (N=39) * Chi^2^ test: non-supplemented group (N=86) vs supplemented groups together (2+3) (N=39). Fischer's exact test: non-supplemented group (N=86) vs supplemented groups together (2+3) (N=39).

### Serum progesterone measurement

The mean serum progesterone concentration after four doses of VMP was significantly higher in group 1 than in group 2 + 3: 16.27 ± 3.58 ng/ml versus 8.81 ± 1.35 ng/ml (p<0.001) (**Table 1**).

Serum progesterone was measured again on FET day and on pregnancy test day. In group 1 (n=86 cycles), results on FET day were available for 56 cycles and was <11ng/ml in three (3/56, 5.4%). On pregnancy test day, results were available for 72 cycles (72/86, 83.7%) and progesterone concentration was <11 ng/ml in 13 (13/72, 18.1%). Among these 13 cycles, there were five positive pregnancy tests (5/13, 38.5%): two biochemical pregnancies, two miscarriages, and one ongoing pregnancy.

In group 2 (n=20 cycles), on FET day, serum progesterone concentration was quantified in 18 cycles (18/20, 90.0%; mean value: 16.83± 2.95 ng/ml). On pregnancy test day, serum progesterone concentration was available for all 20 cycles (mean value: 15.03 ± 3.03 ng/ml). Serum concentration was <11ng/ml (9.70 ng/ml) only in one woman who had a miscarriage.

### Reproductive outcomes

The OPR was significantly higher in group 2 + 3 than in group 1 (41.03% versus 18.60%; p= 0.008) (**Table 2**). The OPR was 45% in group 2 (subcutaneous progesterone supplementation) and 36.84% in group 3 (oral dydrogesterone supplementation).

**Table 2:** Comparison of reproductive outcomes between the groups according to the progesterone supplementation.

	Group 1 (n = 86)	Group 2+3 (n = 39)	P-value	Group 2 (n = 20)	Group 3 (n = 19)
Implantation rate *(%)*	25.00 ±40.40	42.30 ±46.65	0.038°	47.50 ±47.22	36.84 ±46.67
Positive pregnancy rate (%)	44.19	53.85	0.316*	60.00	47.37
Biochemical pregnancy rate (%)	13.95	5.13	0.147	5.00	5.26
Clinical pregnancy rate (%)	25.58	41.03	0.082*	45.00	36.84
Ongoing pregnancy rate *(%)*	18.60	41.03	0.008*	45.00	36.84
Miscarriage rate (%)	38.46	15.79	0.370°	18.80	12.50

Group 1: non-supplemented; Group 2: subcutaneous progesterone supplementation; Group 3: oral dydrogesterone supplementation.

P, progesterone.

°Wilcoxon's test: non-supplemented group (N=86) vs supplemented groups together (2+3) (N=39) * Chi^2^ test: non-supplemented group (N=86) vs supplemented groups together (2+3) (N=39). Fischer's exact test: non-supplemented group (N=86) vs supplemented groups together (2+3) (N=39).

The positive pregnancy rate was slightly higher in group 2 + 3 than in group 1 (53.85% versus 44.19%; p= 0.316). Biochemical pregnancies and miscarriages tended to be more frequent in group 1 than group 2 + 3 (13.95% versus 5.13%, p=0.147, and 38.46% versus 15.79%, p=0.182, respectively).

### Logistic regression analysis

A logistic regression analysis was performed in which OPR was the dependent variable and the covariates were woman’s age, BMI, smoking, previous deliveries, rescue protocol, number of transferred embryos in previous attempts, endometrial thickness, day of embryo transfer, and number of embryos transferred in the cycle. Only age, number of transferred embryos, and rescue protocol were significantly different between group 1 and group 2 + 3 by univariate analysis (p<0.1) (**Table 3**). After adjustment for woman’s age and number of transferred embryos, only rescue protocol remained significantly associated with higher OPR in the multivariate analysis (adjusted OR = 3.25, 95% CI [1.38 – 7.68], p=0.007).

**Table 3:** Logistic regression analysis to identify factors associated with ongoing pregnancy rate.

Covariate	Univariate (N=125)			MULTIVARIATE		
	OR	95% Cl	*P*	OR	95% Cl	*P*
Women's age	0.91	[0.83 -1.00]	0.053	0.90	[0.82-0.99]	0.039
Body mass index (kg/m^2^)	1.02	[0.91-1.16]	0.704			
Smoking			0.214			
No	1					
Yes	0.46	[0.13-1.70]				
Previous delivery			0.282			
No	1					
Yes	1.56	[0.69-3.52]				
Progesterone supplementation			0.009			0.007
No	1			1		
Yes	3.04	[1.32-7.04]		3.25	[1.38-7.68]	
Number of embryos transferred in	0.89	[0.69-1.15]	0.359			
previous transfers						
Endometrial thickness						
before progesterone	0.93	[0.74-1.17]	0.520			
start (mm)						
Day of embryo transfer			0.554			
Day 4	1					
Blastocyst	0.76	[0.32-1.84]				
Number of embryos transferred	2.05	[0.90-4.67]	0.089			

OR, Odd ratio; Cl, confidence interval.

## Discussion

This retrospective study showed that a rescue protocol (subcutaneous progesterone or oral dydrogesterone) in women undergoing HRT FET cycles with preimplantation genetic testing with serum progesterone concentration <11 ng/mL after four doses of VMP allows maximizing the reproductive outcomes (higher OPR) compared with patients with adequate progesterone concentration.

To the best of our knowledge this is the first study to offer a rescue protocol early after progesterone introduction in a HRT cycle. Progesterone plays a crucial role in the secretory transformation of the endometrium and in the opening of the implantation window. Many studies showed a negative impact of low serum concentration of progesterone in the mid-luteal phase on the reproductive outcomes (6, 7, 9, 12–17). Therefore, we hypothesized that early implementation of a rescue protocol to increase its concentration should optimize endometrial receptivity and maximize reproductive outcomes. However, a recent study showed that endometrial receptivity, assessed by Endometrial Receptivity Analysis, was associated with higher endometrial progesterone levels. Conversely, no correlation was found between endometrial and serum progesterone concentrations or between endometrial receptivity and serum progesterone concentration (31). Moreover, most studies on rescue protocols showed an effectiveness of progesterone adjustment started later in the cycle (i.e. blastocyst transfer day or the day before) (5, 20, 21). This suggests that the implantation window is not the only criterion for success and that the increase of reproductive outcomes observed with the rescue protocol might also be linked to other progesterone effects (32, 33). Indeed, progesterone can help to support pregnancy thanks to its muscle relaxing effect that prevents the onset of uterine contractions (33). Moreover, it is involved in the adaptive immune response underlying the maternal-fetal tolerance necessary for pregnancy development (33). As suggested by de Ziegler and al., progesterone role in reproductive outcomes depends on its uterine and also extra-uterine actions (32). Besides the high uterine progesterone concentration conferred by VMP, it seems that the increase in serum progesterone concentration further enhances the chances of success, demonstrating the importance of its extra-uterine effects (32).

Our study also showed that in group 2 (subcutaneous progesterone) the rescue protocol restored progesterone levels, as indicated by the progesterone measurements on FET day (>11ng/ml in all cycles) and pregnancy test day (>11ng/ml in 95% of cycles). In group 3 (oral dydrogesterone), these data are not available because dydrogesterone and its metabolite 20α-dihydroprogesterone can only be measured with specific, not routinely used laboratory techniques (30). This result in group 2 is in agreement with the 98% of correction rate found by Alvarez et al. after addition of subcutaneous progesterone (25 mg) in patients with progesterone <10.6ng/ml (21). However, the pharmacokinetic profiles of subcutaneous and vaginal progesterone are different. Serum progesterone concentration peak is observed earlier and is higher after subcutaneous injection than with the vaginal form, and steady state is reached within 4 days of treatment (34). During the first 4 days after introduction of subcutaneous progesterone, the serum concentration is influenced by the interval between blood sampling and last injection (34). Moreover, most studies that reported cut-off values of serum progesterone to define the “low serum progesterone concentration” used vaginal progesterone. Thus, the optimal threshold with injectable progesterone or combined forms remains to be determined (12, 35).

This is also the first study to use oral dydrogesterone as a rescue protocol in HRT despite its large use in assisted reproductive technologies. The efficacy of oral dydrogesterone for LPS in fresh IVF cycles is well established (26–29). In a meta-analysis published in 2020 that included two phase III multicenter clinical trials comparing the efficacy of dydrogesterone 30mg daily versus VMP 600 mg daily (26) or 8% VMP gel 90mg daily (27) in fresh IVF cycles, Griesinger et al. found a significantly higher OPR and higher live birth rate with oral dydrogesterone than VMP (28). The reasons of oral dydrogesterone better efficacy are not fully understood. It could be related to its specific features: high oral bioavailability and high specificity for progesterone receptors (28). Limited data are available on its use in FET HRT cycles. A meta-analysis by Barbosa et al. that included 11 studies and 4061 patients showed similar live birth rates, OPR and clinical pregnancy rates in patients undergoing HRT with LPS with dydrogesterone (20 mg to 40 mg per day) or with VMP (600mg to 800 mg per day) (29). Data are lacking also α-dihydroprogesterone concentration and reproductive outcomes. Recently, some authors reported that their low serum levels on embryo transfer day during HRT cycles with dydrogesterone alone were associated with lower OPR (30). Furthermore, as inter-individual variations in serum levels have been observed, monitoring dydrogesterone levels could be interesting (30). In a prospective cohort study, Lan N. Vuong et al. compared the reproductive outcomes of two LPS protocols in HRT: vaginal progesterone alone (800 mg per day) or vaginal progesterone plus dydrogesterone (10mg twice per day). Treatments were given sequentially in two different periods and whatever the serum progesterone concentration. Live birth rate was higher in the progesterone + dydrogesterone group than progesterone group, but the difference was not significant [46.3% versus 41.3% (RR 1.12, 95% CI 0.99–1.27, p = 0.06; multivariate RR 1.30 (95% CI 1.01–1.68), p = 0.042)] (36). Miscarriage rate at <12 weeks was significantly lower in the progesterone + dydrogesterone group, in line with our results and supporting the use of dydrogesterone as rescue strategy in HRT (36).

Although a multiple regression analysis was performed, the main limitations of our study were the retrospective design and the small sample size. Moreover, patients could choose the progesterone supplementation method. Their choice could have been influenced by the price of subcutaneous progesterone (not reimbursed in France) and the information we gave (i.e. subcutaneous progesterone already used in rescue protocols with demonstrated effects, unlike dydrogesterone) (5, 20–22). In addition, we used a serum progesterone cut-off of 11ng/ml to define an insufficient LPS based on previous studies (9, 13). However, there is no consensus on the optimal cut-off and timing of measurement. The ideal progesterone concentration seems to range between >8 and 15 ng/ml (6, 7, 9, 13–16). When taking into account only studies using vaginal progesterone and blastocyst transfer, Melo et al. found that higher progesterone level was associated with higher OPR and live birth rate and lower miscarriage risk when using progesterone thresholds <10ng/ml and between 10 and 20ng/ml, but not with progesterone thresholds >20ng/ml because of a large interstudy heterogeneity (16). We did not demonstrate that patients with progesterone levels <11 ng/ml without rescue protocol had lower reproductive outcomes compared with those with progesterone >11ng/ml. Nevertheless, the evidence of decreased reproductive outcomes in patients with low progesterone level around embryo transfer seems to be robust in the literature. Consequently, not supplementing these patients does not seem ethical (16, 17). More studies are needed to define a minimal progesterone threshold to maximize the chance of success.

Several studies focused on the effectiveness of a rescue protocol in HRT cycles with low serum progesterone concentration, but they were heterogeneous in terms of blood sampling time, serum progesterone threshold, and type of rescue protocol (5, 9, 20–22). The main rescue protocols described in the literature are summarized in **Table 4**. As vaginal absorption of progesterone seems to reach saturation, VMP dose increase does not improve OPR in patients with low progesterone level during HRT (9, 10). Therefore, alternative rescue protocols include the addition of subcutaneous progesterone (25mg daily) on the day before embryo transfer or at embryo transfer day (day 3 embryos or blastocysts). Reproductive outcomes were similar between patients with adequate serum progesterone levels and patients with low serum progesterone levels who followed these protocols (5, 20–22). In our study, the rescue protocol after four doses of 400 mg VMP allowed almost doubling the OPR compared with group 1 (without rescue due to adequate progesterone concentration). However, OPR in group 1 was low (18%) compared with other studies. This could be explained by the higher biochemical pregnancy and miscarriage rates in this group. Although data are heterogeneous, the OPR in patients with adequate progesterone levels around embryo transfer day varied between 33% and 54% (5, 9, 20–22). Similarly, their miscarriage rate was lower, but different definitions of miscarriage were used. In a recent randomized clinical trial, Devine et al. compared three routes of progesterone administration as LPS in HRT: intramuscular only, intramuscular + vaginal, and vaginal only. The vaginal arm had to be prematurely stopped due to their higher rate of early miscarriage (50% versus 26% in the intramuscular + vaginal group and 33% in the intramuscular group; p <0.0001), although the positive pregnancy rate was comparable among arms (23). Our results are in line with those by Devine et al., although we used a different dose of progesterone by the vaginal route (200mg twice daily vs 800 mg/day in our study), and a different miscarriage definition (pregnancy loss after positive pregnancy test), and they did not measure serum progesterone concentration (23). Vuong et al. found a significantly lower miscarriage rate in patients supplemented with dydrogesterone + VMP than with VMP alone (36). Despite a careful methodology, we can hypothesize that our results in group 1 can be explained by the small sample size. When we checked progesterone concentration on pregnancy test day, we found that it was <11ng/ml in ~18% of patients in group 1. On embryo transfer day, it was <11ng/ml only in three patients of group 1, but it was not measured in 30/86 women, suggesting that their number could have been bigger. These results are not in line with pharmacokinetic studies (18), but could explain the higher biochemical pregnancy rate and miscarriage rate in this group. This can question the early progesterone monitoring after four doses of vaginal progesterone to assess the need of supplementation, especially because studies on “rescue protocols” showed the effectiveness of a late supplementation (around embryo transfer day) (5, 9, 20–22). In fact, there is no consensus on the ideal time for progesterone monitoring and supplementation, and on the best serum progesterone threshold. We may ask whether our threshold of 11 ng/ml is adequate or whether we should choose a higher threshold to reduce the risk of having patients with low serum progesterone concentration at embryo transfer/pregnancy test day. In addition, it is important to note that the patients in the study received PGT-SR or PGT-M, but that pre-implantation genetic testing for aneuploidy (PGT-A) is not allowed in France: the risk of aneuploidy at the time of transfer is therefore the same as in the general population, which may also explain our miscarriage and biochemical pregnancy rates. For few months, some practitioners in the center introduced progesterone supplementation if serum progesterone was <11ng/ml on embryo transfer day. These cycles were excluded from the study analysis for protocol violation, but their OPR was good, similar to what observed in the supplemented group. This may have falsely decreased OPR in group 1. Therefore, our findings should be interpreted with caution because of the lower than expected OPR in group 1 (control group).

**Table 4:** Rescue protocols described in previous studies in function of serum progesterone concentration in hormone replacement therapy cycles.

Study	Design	Population (n)	LPS	Embryo transfer day	Progesterone threshold and day of measurement	Rescue protocol and starting day	Results
Cedrin-Durnerin et al. 2019 (9)	Retrospective cohort	227 FET cycles	VMP 200mg, three times/day	Day 3 or blastocyst stage	<10ng/ml On ETday (n=85 FET cycles)	Switch to VMP 400mg three times/day on ET day	OPR and LBR lower when progesterone <10ng/ml despite the rescue: 17% vs 33% (p=0.01) and 31% vs 17% (p=0.02)
Yarali et al. 2021 (20)	Case-control study	160 patients	Vaginal progesterone gel 8%, twice/day	Blastocyst stage	<8.75ng/ml On the day before FET (n=40 patients)	Addition of subcutaneous progesterone 25mg once/day on the day before ET	OPR comparable when progesterone >8.75ng/ml: 48.3% vs 50.0% in the rescue group (p=0.858)
Alvarez etal. 2021 (21)	Prospective cohort	574 FET cycles	VMP 200mg/8h	Blastocyst stage	<10.6ng/ml On the day before FET (n=226 FET cycles)	Addition of subcutaneous progesterone 25mg once/day on the day before ET	Similar outcomes between groups: OPR 49.4% when progesterone >10.6ng/ml vs 53.6% with rescue, LBR 49.1% vs 52.3%
Labarta et al. 2022 (5)	Retrospective cohort	1849 patients	VMP400mg/12h	Blastocyst stage	<9.2ng/ml On ET day (n=550 patients)	Addition of subcutaneous progesterone 25mg once daily on ETday	Similar outcomes between groups: OPR 45.2% when progesterone >9.2 ng/ml vs 44.9% with rescue, LBR 45% vs 44.9%
Ozcan et al. 2022 (22)	Prospective cohort	222 FET cycles	100mg vaginal progesterone tablet, twice/day + 250mg intramuscular progesterone	Day 3 or blastocyst stage	<10ng/ml On ET day (n=65 FET cycles)	Addition of subcutaneous progesterone 25mg once/day on ET day	Similar outcomes between groups: OPR 55.4% when progesterone >10ng/ml vs 61.5% with rescue (p=0.4), OPR 78.2% per pregnancy vs 72.5% (p=0.5)

LPS, luteal phase support; FET, frozen embryo transfer; VMP, vaginal micronized progesterone; ET, embryo transfer; OPR, ongoing pregnancy rate; LBR, live birth rate.

In conclusion, in patients undergoing HRT FET cycles for preimplantation genetic testing, a rescue protocol with subcutaneous progesterone or oral dydrogesterone in case of low serum progesterone concentration level after four doses of VMP seems to increase OPR compared with patients with adequate serum progesterone. A randomized clinical trial is needed to confirm our results and determine the best rescue protocol and the best time for its introduction.

## Data availability statement

The original contributions presented in the study are included in the article. Further inquiries can be directed to the corresponding author.

## Ethics statement

This study was approved by the Institutional Review Board (approval number IRB-MTP_2022_04_202201084).

## Author contributions

BDB is the main author, BDB collected all the data, wrote the manuscript and designed the figures, working under the direction of TA. The methodology was elaborated in association with TA and CM. CM performed statistical analysis. NR, SB and SBD completed the screening on full text, assessed the quality, and revised the manuscript. All authors contributed to the article and approved the submitted version

## Conflict of interest

The authors declare that the research was conducted in the absence of any commercial or financial relationships that could be construed as a potential conflict of interest.

## Publisher’s note

All claims expressed in this article are solely those of the authors and do not necessarily represent those of their affiliated organizations, or those of the publisher, the editors and the reviewers. Any product that may be evaluated in this article, or claim that may be made by its manufacturer, is not guaranteed or endorsed by the publisher.
